# Exploring BCG vaccination as a novel approach to prevent recurrent herpes labialis

**DOI:** 10.1016/j.eclinm.2023.102279

**Published:** 2023-10-17

**Authors:** Wenping Gong, Jingli Du, Li Zhuang, Xueqiong Wu

**Affiliations:** a Beijing Key Laboratory of New Techniques of Tuberculosis Diagnosis and Treatment, Senior Department of Tuberculosis, The Eighth Medical Center of PLA General Hospital, Beijing, 100091, China; b Hebei North University, Zhangjiakou, 075000, Hebei, China

According to WHO, approximately 3.7 billion individuals under the age of 50 years (67%) carry herpes simplex virus type 1 (HSV-1), the primary cause of herpes labialis.^1^ Although medication can alleviate symptoms, it cannot cure the infection and recurrence may occur. The Bacillus Calmette-Guérin (BCG) vaccine is the only approved attenuated vaccine for tuberculosis prevention. Research suggests that BCG vaccination induces three types of non-specific immune responses: trained immunity, heterologous immunity, and anti-inflammatory effects.^2^^,^^3^ These immune responses have been used to prevent and treat a range of diseases, including HSV-1, human papillomavirus, and COVID-19^3^, ^4^, ^5^, ^6^ (Fig. 1).

**Fig. 1:**
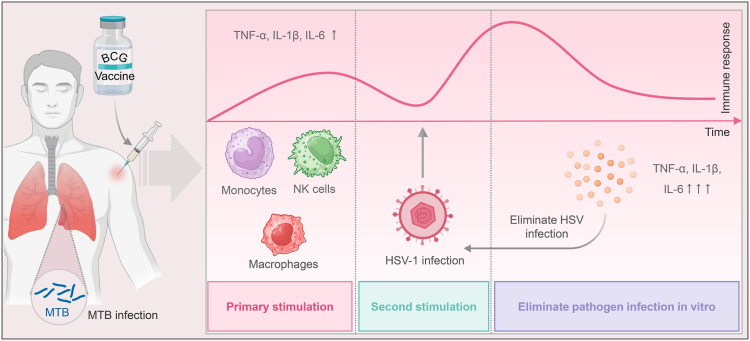
**Non-specific immune response induced by BCG**. BCG immunisation or MTB infection of the human body induces an increase in gene rearrangement and metabolism of intrinsic immune cells, such as macrophages, NK cells, and monocytes, resulting in an increase in the secretion of cytokines, such as TNF-α, IL-1β, and IL-6. During re-infection with other pathogens like HSV-1, these trained innate immune cells will rapidly secrete a large amount of TNF-α, IL-1β, IL-6, and other cytokines to kill and eliminate invading pathogens.

In *eClinicalMedicine*, Laure F Pittet and colleagues published their phase 3 clinical trial that was conducted across multiple countries to assess the impact of BCG vaccination on recurrent herpes labialis.^7^ 84 patients with frequent episodes of herpes labialis were enrolled and randomly assigned to either the BCG group (n = 38) or the control group (n = 46). The results demonstrated that, over a 1-year follow-up period, the BCG group experienced reduced duration, frequency, severity, and impact on quality of life related to recurrent herpes labialis than did the control group. Furthermore, in the BCG-immunised individuals, the time to the first recurrence was extended by 1.55 months compared with the control group (p = 0.02), indicating potential benefits of BCG vaccination for individuals with herpes labialis (p = 0.003). Subgroup analysis revealed that the benefits of BCG vaccination were primarily observed in male participants. A 2020 systematic review also demonstrated the benefits of BCG vaccination for 78% of adult patients with recurrent genital or herpes labialis, with 37% experiencing long-term remission and a reduction in outbreak frequency or severity by 41%.^4^ Although these results differ from a 1985 observational clinical trial by J M Douglas and colleagues—which found that BCG vaccination did not significantly reduce the recurrence rate of genital herpes or prolong the time to the first recurrence^8^—discrepancies may be attributed to variations in sample size, BCG vaccine types, dosage, administration methods, and the immunological status and genetic backgrounds of vaccine recipients.

A cautious interpretation of the clinical trial results is necessary, and these limitations are acknowledged by the study authors in their manuscript. Firstly, the study had a relatively small sample size, with only 38 individuals receiving BCG vaccination and 46 individuals in the non-vaccinated group. Secondly, the participants’ immune status during the study period was not taken into consideration. If participants had a compromised immune system during the study, BCG recipients may not have regained their antiviral immunity, leading to reactivation of latent viruses and subsequent recurrence of herpes labialis. Therefore, it is recommended to assess the quantity of immune cells and immune function in the participants before and after BCG vaccination. Thirdly, despite the authors' presentation of the BCG vaccination history, tuberculosis (TB) history, and tuberculin skin test (TST) results obtained through questioning in Table 1 in Pittet, L.F′ study,^7^ participants were not screened for *Mycobacterium tuberculosis* (MTB) infection status using either the TST or interferon gamma release assay (IGRA) methods. This lack of screening may have obscured the participants' MTB infection status at the time of enrollment. For example, a study conducted in 1992 to investigate the effectiveness of a single intradermal injection of BCG in Tine test-negative individuals excluded factors such as latent tuberculosis infection (LTBI) and reinoculation of BCG.^9^ 109 patients with herpes simplex virus, who tested negative for the tuberculin Tine test, received the BCG vaccine, with their own condition serving as the control. Following vaccination, all patients remained free of herpes for a minimum of 4–6 months. During follow-up, 21 patients (19%) remained free of herpes after 3 years and 10 patients (9%) did not experience outbreaks for over 6 years.^9^ These findings suggest that screening participants for MTB infection status at enrollment in clinical trials evaluating the protective effect of BCG against herpes labialis recurrence is useful in improving the accuracy of clinical trial results. We strongly advise that any clinical trial aiming to assess the immunoprotective effect of BCG should rigorously screen participants using the TST method in low TB prevalence areas and the IGRA method in high TB prevalence areas. It is important to note that MTB and other non-tuberculous mycobacterial infections can elicit non-specific immune responses similar to BCG, potentially influencing the evaluation of BCG treatment efficacy.^10^

Nonetheless, the study by Pittet and colleagues is a unique and innovative contribution to the literature. It compared the protective effect of BCG vaccination against recurrent herpes labialis between sexes and suggested an increased risk of primary cold sore occurrence following BCG vaccination.^7^ The results imply that BCG-induced non-specific immune responses have the potential to enhance the body's immune response, but they may also exacerbate existing inflammatory or immune-related diseases. Therefore, when utilising BCG-induced non-specific immune responses for disease prevention or treatment, the population's immune state and underlying diseases should be considered. Well-designed studies are necessary to avoid potential adverse effects and meticulous evaluation and analysis should be conducted, maximising the beneficial effects of the various immunological responses induced by BCG.

## Contributors

WPG and JLD conceptualised this work. WPG and LZ analysed the data. WPG and JLD were responsible for writing the work, with review and revisions by WPG and XQW. WPG and XQW also acquired funding. All authors reviewed and approved the final manuscript.

## Declaration of interests

XQW has received research funding from the National Key Research and Development Program of China (grant No. 2022YFA1303503), and payments were made to her institution. WPG has received research funding from the Eighth Medical Center of PLA General Hospital (grant number MS202211002), and payments were made to his institution. XQW, WPG, and JLD currently serve as executive members of the Chinese Antituberculosis Association without any payments.
